# Chromatic induction and retinal image motion

**DOI:** 10.1177/03010066251409616

**Published:** 2026-01-07

**Authors:** Y. Howard Li, Michele Rucci, Borja Aguado, Cristina M. Maho, Martina Poletti, Eli Brenner

**Affiliations:** 1 Center for Visual Science, 460225 University of Rochester, Rochester, NY, USA; 2 GRAD Atenció a la Diversitat, Psychology Department, Faculty of Education, Translation, Sports and Psychology, 16783 Universitat de Vic-Universitat Central de Catalunya, Vic, Spain; 3 Department of Human Movement Sciences, 1190 Vrije Universiteit Amsterdam, Amsterdam, The Netherlands

**Keywords:** colour vision, chromatic induction, ocular drift, simultaneous colour contrast

## Abstract

As the eyes drift across a scene, borders between surfaces slide across the retina. Consequently, near borders’ edges, parts of the retina that have adapted to the light at one side of the border are exposed to the light at the other side of the border. Such changes in exposure might increase the judged contrast. Retinal image motion might therefore contribute to chromatic induction, the influence that adjacent colours have on a surface's apparent colour, by increasing the apparent colour contrast. We conducted two experiments to evaluate this possibility. The experiments examined how artificially increasing or decreasing the extent to which certain surface borders shift across the retina influences the perceived colour. Neither increasing nor decreasing the extent to which selected borders shift across the retina had a substantial influence on the perceived colour. This implies that chromatic induction does not arise from overestimating the contrast between adjacent surfaces when small eye movements shift the border between those surfaces across the retina.

## Introduction

A surface's apparent colour depends on the chromaticity of neighbouring surfaces as well as on the surface's own chromaticity (Brenner et al., 2007a, 2007b; Hansen & Gegenfurtner, 2005; Shevell & Wei, 1998). When using relatively simple displays, the influence of neighbouring surfaces is often referred to as simultaneous colour contrast or chromatic induction (Bachy & Zaidi, 2016; Hurlbert & Wolf, 2004; Jameson & Hurvich, 1961; Singer & D’Zmura, 1994; Walraven, 1976; Ware & Cowan, 1982). Understanding the mechanisms of chromatic induction might provide a useful step to understanding human colour vision.

The prevalent hypothesis is that chromatic induction emerges when processing the chromatic contrast between adjacent surfaces. The visual system exploits edge contrasts to infer a surface's chromaticity because such contrasts remain relatively constant under changes in illumination (Land, 1983; Land & McCann, 1971), at least as long as the surfaces are coplanar (Bloj, Kersten & Hurlbert, 1999; Boyaci, Doerschner & Maloney, 2004). If this account is correct, chromatic induction arises from failures to correctly attribute colour contrasts between surfaces to the chromaticities of those surfaces when judging a surface's colour (Foster & Reeves, 2022; Land, 1986; Nascimento et al., 2004). This is particularly likely to occur in very simple scenes, such as the typical scenes used to illustrate chromatic induction.

An alternative hypothesis is that chromatic induction emerges from the contrast across edges being overestimated (Ekroll & Faul, 2012). Such overestimation might be related to eye movements shifting the image across the retina. Even during steady fixation, the image continually moves on the retina because of small eye movements (ocular drifts and micro-saccades). Images tend to fade when stabilized on the retina for prolonged periods (Krauskopf, 1963), and recent evidence shows that, during the brief intervals of natural intersaccadic fixation, the temporal signals resulting from small eye movements help to encode spatial information (Rucci & Poletti, 2015; Rucci & Victor, 2015; Rucci, Ahissar & Burr, 2018). Specifically, with achromatic stimuli, the incessant intersaccadic motion of the eye enhances sensitivity to high spatial frequencies. It does so in proportion to the strength of the resulting luminance modulations (Intoy et al., 2024), and the full spatiotemporal contrast-sensitivity function is well predicted by the spatial information conveyed by fixational modulations (Casile et al., 2019). Consistent with this hypothesis, neuronal responses to edges between surfaces of different colours increase when those edges are swept across the retina, simulating the effect of eye movements (Ennis et al., 2014).

However, sharp transitions at surfaces’ edges do not appear to be critical for chromatic induction, because blurring such edges has little impact (Bachy & Zaidi, 2016). So, it may not specifically be the contrast at surfaces’ edges that is misjudged. Eye movements might influence the locally judged chromaticity within the target surface. Colour vision is known to rely less exclusively on edge contrast than luminance does (Wachtler & Wehrhahn, 1997). Nevertheless, small eye movements that are made during fixations may play a similar role to the one described above. Without eye movements, the saturation of the local chromaticity would probably be underestimated due to retinal adaptation. As the eyes move, parts of the surface of interest move onto parts of the retina that are adapted to the neighbouring colours, reducing such adaptation. But this would also bias the perceived hue (Cornelissen & Brenner, 1995; D’Zmura & Lennie, 1986; Ennis et al., 2014). Cones adjust their sensitivity based on recent stimulation (von Kries, 1905), and even brief adaptation can alter subsequent colour appearance (Cornelissen & Brenner, 1995; Rinner & Gegenfurtner, 2000). If the area surrounding a surface of interest is biased towards a certain colour, as it is in the typical chromatic induction display, the parts of the retina that are exposed to the surface of interest will often have recently been exposed to the colour in the surrounding and therefore make the surface appear to have the complementary chromaticity. Thus, a combination of eye movements and local adaptation could produce chromatic induction without requiring edges to play a critical role.

Several authors have proposed that eye movements might determine how the surrounding is considered when judging the colour of a target in a scene (e.g., Cornelissen & Brenner, 1995; D’Zmura & Lennie, 1986; Lee et al., 2012). Moreover, gaze is known to modulate judgments of a surface's colour: where gaze was directed immediately before making a colour judgment (Cornelissen & Brenner, 1995) and when making such judgments (Brenner et al., 2007a; Golz, 2010) alters the perceived hue. Eye movements also specifically modulate chromatic induction (Cornelissen & Brenner, 1991), although inter-individual differences in chromatic induction could not be explained by differences in gaze patterns (Granzier et al., 2012).

Regardless of how eye movements influence chromatic induction, if they play a substantial role we predict that chromatic induction strength will scale with the magnitude of retinal slip. If chromatic induction primarily arises from misattribution of the contrasts across edges, then the magnitude of retinal slip should have little effect. Thus, in the present study, we examine whether manipulating the amount of retinal motion of selected borders influences chromatic induction. We do so by adding jitter to such borders or stabilizing the image on the retina, mimicking variations in fixational eye movements

## Methods

**Figure 1.**
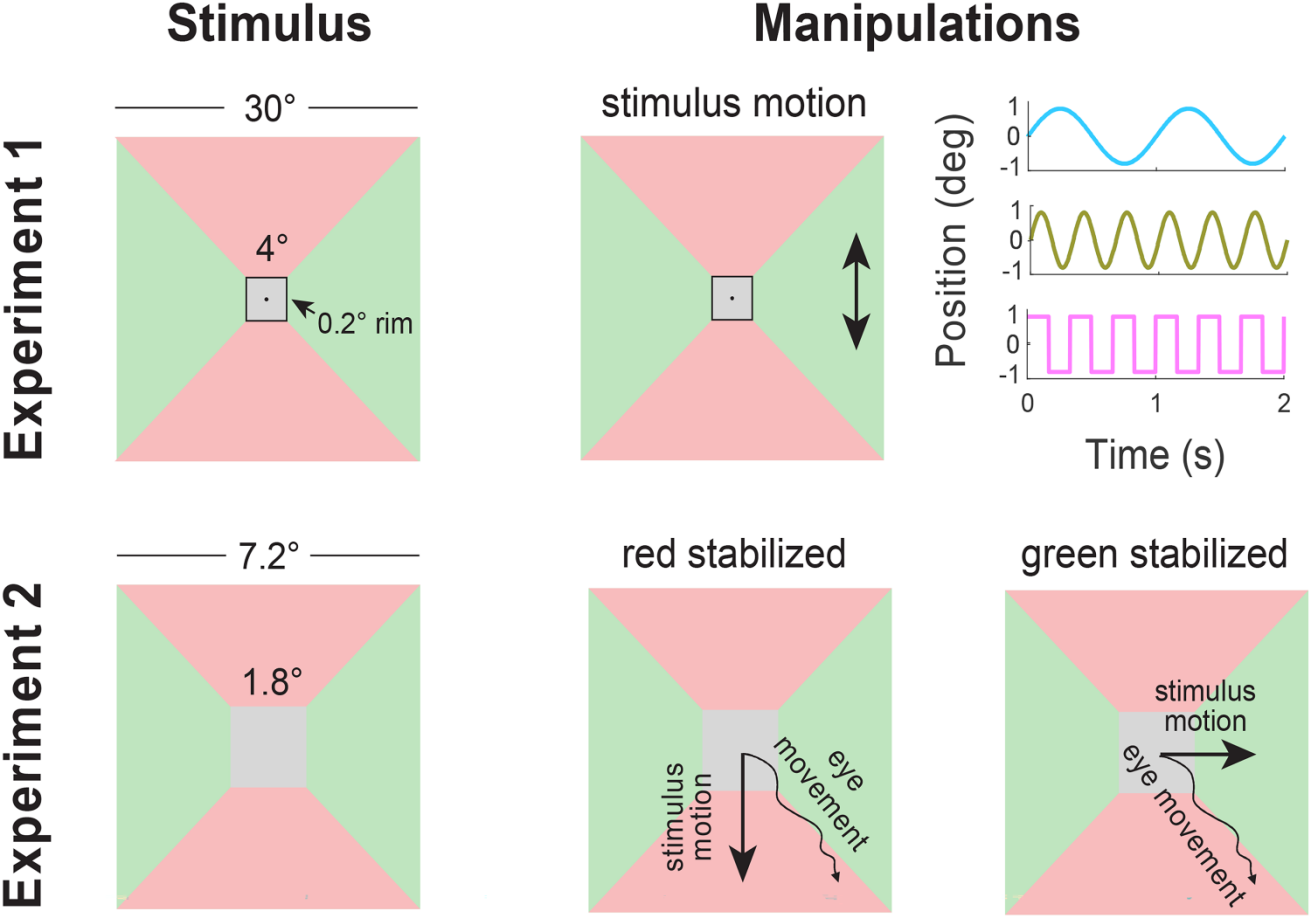
Schematic representation of stimuli with red surfaces above and below the target, and green surfaces to its left and right. In Experiment 1 (top row), there could be a rim between the target and the surrounding surfaces. The whole image except a central fixation point could move vertically (arrows in central panel) or horizontally (not shown). The motion followed one of the three patterns shown in the right panel (1 Hz sinusoidal, 3 Hz sinusoidal, 3 Hz square wave). In Experiment 2 (bottom row), the image followed either the vertical (central panel) or the horizontal (right panel) component of any eye movement.

In separate experiments, we either increased the changes in retinal exposure by jittering the stimulus on the display (Experiment 1), or decreased them by stabilizing the image on the retina (Experiment 2). If these manipulations clearly influence the perceived colour, we can conclude that a combination of retinal adaptation and eye movements contributes to chromatic induction. In both experiments, the colour of the surrounding was either uniform or divided into four sections so that the regions above and below the square target surface had one colour, and the regions to its left and right had another (Figure 1). Uniform surroundings were included to verify that chromatic induction is not altogether disrupted by jittering or by partially stabilizing the images, or by some other aspect of the specific tasks and procedures that we used in our experiments. Placing the target surface between two red surfaces in one direction and two green surfaces in the orthogonal direction allowed us to either increase or decrease the extent to which the target's border with either the red or the green surface shifted across the retina, without changing the overall instantaneous (spatial) colour contrasts within the image. The increase was achieved by jittering the images in one of the two directions (Experiment 1; see Movie 1). The decrease was achieved by stabilizing them on the retina in one of the two directions (Experiment 2).

### Methods of Experiment 1

In Experiment 1, the participants’ task was to adjust the target surface's colour to look neither reddish nor greenish (grey) by moving the computer mouse. Images were presented at 120 Hz on a computer screen that was 57 cm from the subject so that 1 cm is about 1 degree of visual angle. The screen was 40 by 30 cm (1280 by 768 pixels). It was calibrated with a Minolta CS-100A Chroma Meter (Minolta Camera Co. LTD., Japan). The target surface was a 4° by 4° square. The surrounding (a 30° by 30° square) was either directly adjacent to the target (rim width of 0°) or separated from the target by a black rim of 0.2° or 0.6°. The rims were introduced because they are known to reduce the influence of the surrounding colour (Brenner & Cornelissen, 1991). If the rims reduce the influence of the surrounding because they reduce the extent to which the same part of the retina is successively exposed to the target surface and to the surround as the eyes drift during fixation, or at least increase the time between exposure to the different surfaces, the influence of such a rim might be smaller when the image jitters. So, jittering the image might have a clearer influence on chromatic induction when there is a rim separating the target from the surround. The jitter consisted of the whole image (except for a black fixation point at the screen centre) constantly moving either horizontally or vertically as participants executed the task. The jitter had a displacement amplitude of 0.8° to either side, so a peak-to-peak amplitude of 1.6°.

Seventeen participants with normal colour vision took part in the experiment after being informed of the task and procedures and signing an informed consent form in accordance with the ethical approval. Each participant took part in 9 sessions in which they were exposed to all combinations of the three rim widths (0°, 0.2°, 0.6°) and three kinds of jitter (1 Hz sinusoidal, 3 Hz sinusoidal, 3 Hz square wave). There were 64 trials per session: 8 replications of each combination of the 2 directions of jitter (vertical or horizontal) and 4 kinds of surrounds (uniformly red, uniformly green, both colours present with green at the sides, both colours present with red at the sides). The 9 sessions were presented in random order, as were the 64 trials within each session. Each session took about 5 min to complete.

We selected the two colours for the surround (that we loosely call *red* and *green* for simplicity), and the range of possible colours of the target, by starting with the default grey of the computer (equal values for the three guns) and a luminance of 35 cd/m^2^. The extent to which this shade of grey stimulated the three kinds of cones was determined by converting the CIE*xy* (that was known from the calibration) and the luminance to cone stimulation in the manner described in Appendix A of Granzier et al. (2009). From those cone stimulation values, we increased (or decreased) l-cone stimulation while decreasing (or increasing) m-cone stimulation in a manner that kept the luminance constant (using the inverse conversion and the calibration). We only used colours along this line in colour space. The two values with the highest l-cone and m-cone stimulation were used as red and green surrounds, respectively (CIE*xy* coordinates: [0.337, 0.301] and [0.220, 0.344]). The luminance and s-cone stimulation were the same for all surfaces except the black rim and fixation point. Participants moved the computer mouse laterally to adjust the target's colour along the red-green dimension until it appeared subjectively grey. Once they were content with their setting, they pressed the button of the computer mouse to confirm this, and the CIE values of the selected target colour were saved for later analysis. The next trial then started with a randomly selected colour from within the available range. Participants could take as long as they liked to refine their judgments, but typically made their settings within a few seconds after the first few trials. They were required to maintain their gaze on the fixation point throughout the trial. They were allowed to take short breaks between the sessions.

Since the participants’ settings were restricted to a single curve in colour space, we only report the data of the CIE*_x_* component. We averaged the CIE*_x_* values across the eight replications and across the two corresponding combinations of images and movement directions to obtain four values per session: a value for the uniform red surround, a value for the uniform green surround, a value for when the jitter moved the target's edge across the boundary of the red part of the two-coloured surround, and a value for when the jitter moved the target's edge across the boundary of the green part of the two-coloured surround. To quantify the influence of the surround, we determined the difference between settings for the uniform red and green surrounds, and the difference between settings when the jitter moved the target's edge across the boundary of the red and green parts of the two-coloured surround. These differences were determined for each participant and session. A three-way repeated measures analysis of variance was conducted on these differences with the factors rim width, kind of jitter, and kind of surround (uniform or two-coloured surround).

### Methods of Experiment 2

In Experiment 2 we wanted to present targets for a fixed, short duration, so participants performed a two-alternative forced choice task, indicating whether the target appeared ‘reddish’ or ‘greenish’. We measured the participants’ eye movements and moved the stimulus along with the eye movements to decrease retinal image motion in either the horizontal or vertical direction. Thus, the retinal image was immobilized in the selected direction (either horizontal or vertical), but eye movements in the orthogonal direction (vertical if the selected direction was horizontal; horizontal if the selected direction was vertical) resulted in the usual shifts of the retinal image across the retina in that direction. We used the same kind of stimuli as in the no-rim condition of Experiment 1, but some details were changed to comply with limitations imposed by the eye tracker and design of the experiment.

Images were presented on a fast phosphor CRT monitor (Iiyama HM204DT) at a resolution of 800 × 600 pixels and vertical refresh rate of 200 Hz in a dimly illuminated room. Subjects looked at stimuli from a fixed distance of 126 cm from the monitor, while their head was immobilized by a dental-imprint bite bar and a head-rest. Stimuli were observed monocularly with the right eye. The left eye was patched. Eye movements were measured using a Generation 6 Dual Purkinje Image eye tracker (Fourward Technologies), a system capable of detecting rotations as small as 1 arcminute, as measured by an artificial eye controlled by a galvanometer (Fang et al., 2018; Ko et al., 2016). Eye positions were low-pass filtered at 500 Hz, sampled at 1 kHz and digitally recorded.

Stimuli were rendered using EyeRIS, a custom system for gaze-contingent display control (Santini et al., 2007) and updated on the display within 10 ms from the eye movements. The target surface was a 1.8° by 1.8° cm square. The surrounding was directly adjacent to the target and it extended to a distance of 2.7° from the edge of the target. The rest of the monitor was a shade of blue devised in the same way as the red and green ones, but increasing S-cone stimulation from the monitor's default grey setting. The same shade of blue filled the whole screen between stimulus presentations. Making the background blue in this manner maintains the L to M cone ratio, while avoiding providing a grey reference. Maintaining isoluminance across the whole screen ensures that the changes in retinal stimulation that arise through eye movements are not masked by large changes in luminance when the target appears. Thus, to attenuate changes in retinal stimulation not caused by eye movements (transients at stimulus onset and offset), the screen was completely filled with the above-mentioned shade of blue between trials, and the stimulus contrast was ramped up and down for each trial so that the stimulus appeared and disappeared gradually rather than abruptly. Each target was ramped up and down for 1000 ms and stayed at plateau for an additional 1000 ms. The participants had to press a key to indicate whether the target looked red or green; a two-alternative forced choice judgment.

Three participants with normal colour vision took part in the experiment, again after being informed of the task and procedures and signing an informed consent form, as approved by the Institutional Review Board at Boston University. Each participant took part in several sessions in which they were exposed to eight combinations of stimuli. The trials of the eight combinations of the two directions of stabilization (vertical or horizontal) and four kinds of surrounds (uniformly red, uniformly green, both colours present with green at the sides, both colours present with red at the sides) were randomly interleaved. As in the first experiment, we combined the eight types of trials to obtain four conditions: uniformly red surround irrespective of direction of stabilization, uniformly green surround irrespective of direction of stabilization, two-coloured surround with the red borders with the target stabilized, and two-coloured surround with the green borders with the target stabilized. Only trials with high-quality oculomotor traces were analysed: trials in which subjects made a saccade or micro-saccade, or in which they blinked during stimulus presentation were removed. We plotted the proportion of red responses as a function of the contribution of the red gun to the (constant) luminance of the target, and fit psychometric functions (cumulative normal distributions) to the data for the four kinds of surrounds.

## Results

### Results of Experiment 1

**Figure 2.**
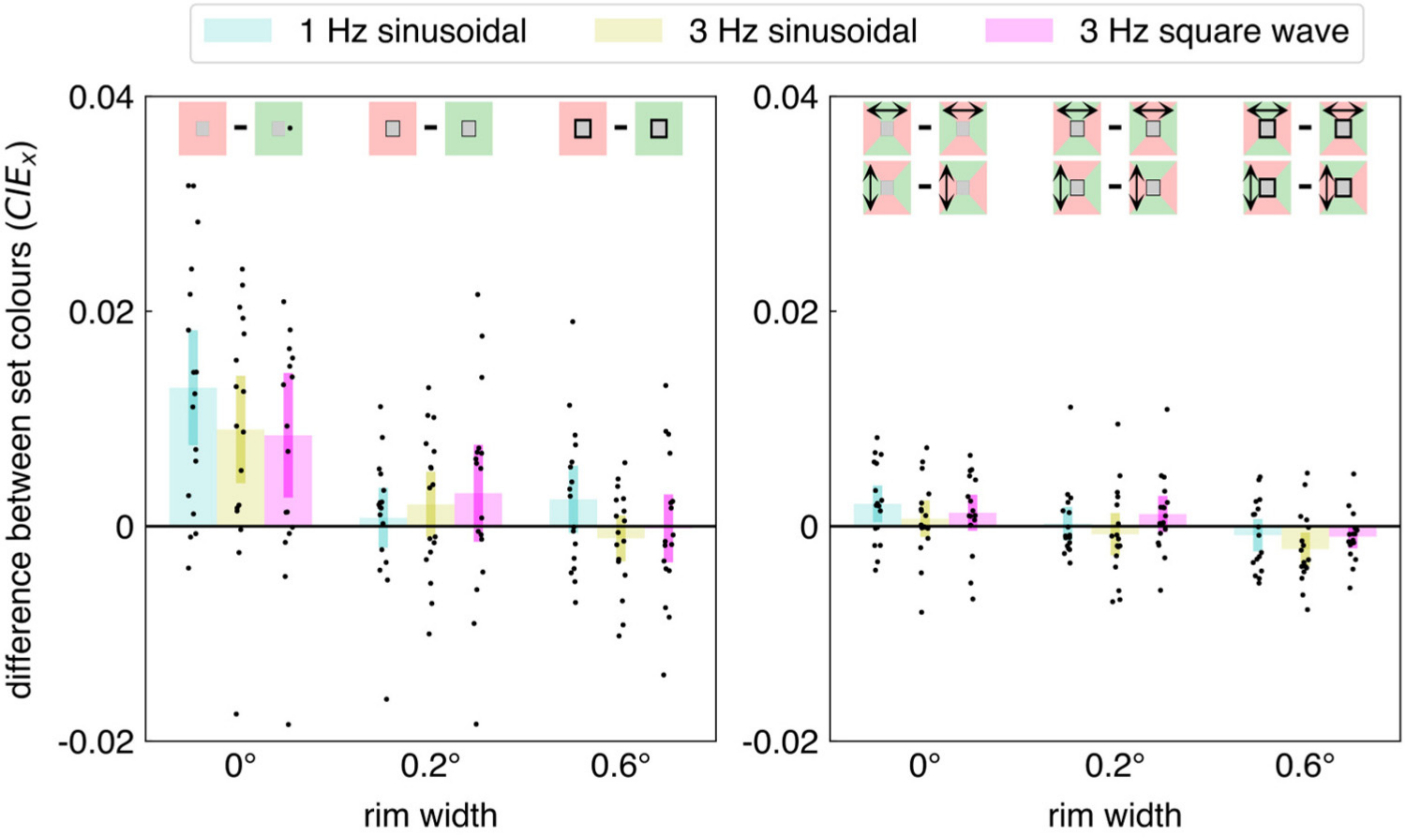
Results of Experiment 1. Each dot represents the difference between one participant's mean set colours in two conditions. In the left panel, the difference is between settings for a red and a green background (see schematic images at top), irrespective of the direction of jitter. In the right panel, it is the difference between settings when the jitter is in the direction of the red part of the background and when the jitter is in the direction of the green part of the background. Each panel shows data for three rim widths and three kinds of jitter (1 Hz sinusoidal, 3 Hz sinusoidal, 3 Hz square wave). The coloured bars show the mean and 95% confidence interval across participants. The differences between the mean set colours are expressed in terms of the shift along the x-axis in the 1931 CIE*_xy_* colour space.

As expected, the uniformly coloured surrounds biased the perceived colour of the target: we found clear chromatic induction. For the first experiment, Figure 2 shows how the three rim widths and three kinds of jitter influence the set target colour for each kind of background (uniform surround or surround with both colours; left and right panel, respectively). The analysis of variance revealed significant effects of rim width (*F*_2,32_ = 27.8 *p* < 0.0001), kind of surround (*F*_1,16_ = 5.81 *p* = 0.028), and interaction between rim width and kind of surround (*F*_2,32_ = 8.78 *p* < 0.001). These presumably all result from the set colour being influenced most by an immediately adjacent uniform background (the chromatic induction that is visible in the leftmost set of three bars of the left panel of Figure 2).

In accordance with previous studies (e.g., Brenner & Cornelissen, 1991), the bias introduced by a uniform, coloured surround was much smaller when it was isolated from the target by even a thin black rim (left panel of Figure 2). Here, this was so despite the image shifting by much more than the rim size. The only other significant effect was an interaction between the kind of jitter and the rim size (*F*_4,64_ = 3.14 *p* = 0.020), for which looking at the data does not provide a simple interpretation. Perhaps this arises because the set colour is influenced most by an immediately adjacent uniform background with slow jitter (leftmost blue bars in both panels of Figure 2; note that slower jitter corresponds with longer adaptation to each colour). Importantly, when there were two colours in the surround (right panel of Figure 2), jittering the image might have slightly increased the influence of the surrounding surface of which the border with the target area was shifted across the retina when there was no rim (rim width 0; possibly consistently positive values), but even if it did, this influence is very small (only a fraction of the influence of a uniform background).

### Results of Experiment 2

**Figure 3.**
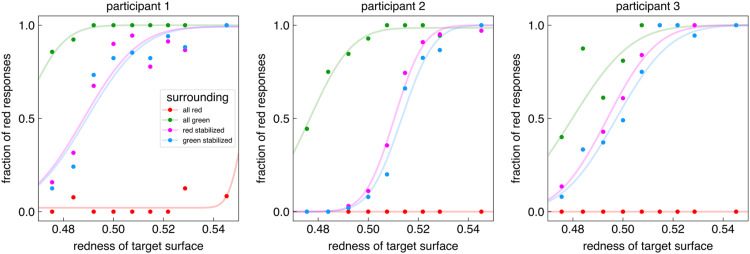
Proportion of red responses as a function of the contribution of the red gun to the luminance of the target for the three participants in the four conditions.

On average, the participants of the second experiment had 783 trials that passed our strict eye movement criteria. Figure 3 shows how the three participants’ responses depended on the kind of background (uniform surround or surround with both colours) and which borders with the background were stabilized. As expected, the uniformly coloured surrounds biased the perceived colour of the target: participants much more frequently reported that the target was red when the background was green than when it was red (*All green* and *All red* curves). Thus, imposing monocular vision with the head immobilized did not eliminate chromatic induction. Importantly, when the surround consisted of two colours and the borders with one of the two colours were stabilized on the retina, the proportion of red responses was hardly influenced by which borders were stabilized and which moved normally on the retina. The blue psychometric curves (green stabilized) are shifted slightly to the right of the purple ones (red stabilized) for all three participants, which is the response that we would expect if eye movements exert an influence, but the shift is very small compared to the large influences exerted by the uniform backgrounds. To evaluate how reliable these shifts are, we used bootstrapping to determine the precision of the mean redness of the target surface at which participants responded ‘red’ half the time (Table 1). For participants 2 and 3, the difference between the means is larger than the sum of the standard deviations, suggesting that the shift is reliable. For participant 1, the shift is in the same direction, but it is smaller and the standard deviations are larger, so we cannot be as certain that this participant's responses were influenced by the stabilization.

**Table 1.** Reliability of the Estimated Redness That Results in 50% Red Responses Based on Bootstrapping.

Participant	Red stabilized	Green stabilized
1	0.4879 ± 0.0018	0.4893 ± 0.0019
2	0.5106 ± 0.0010	0.5134 ± 0.0010
3	0.4938 ± 0.0014	0.4974 ± 0.0016

Values are means and standard deviations of the mean values of cumulative normal distributions fit to 1000 random samples (of the same number of trials as in the data) for each participant and condition.

## Discussion

The aim of this study was to investigate how retinal image motion contributes to chromatic induction. Our results indicate that increasing or decreasing the retinal motion of selected borders had only a very small effect on the perceived colour.

When there was no rim, jittering the background such that edges with one of the two colours shifted more across the retina than edges with the other seems to have influenced the set colour to some extent. The effect was in the anticipated direction, but was only really clear for the slower jitter rate (leftmost blue bar in the right panel of Figure 2; for that condition the 95% confidence interval does not cross zero). The conventional chromatic induction also appears to be largest for the slower jitter, which is consistent with adaptation playing a role in chromatic induction (Cornelissen & Brenner, 1991; Shevell & Wei, 1998). In Experiment 2, where we stabilized selected borders to minimize retinal slip, we again observed a small amount of chromatic induction in the anticipated direction: for all three participants the blue curve is to the right of the purple one. But, although there may be some effect of jittering or stabilizing parts of the stimulus, the effect of selectively increasing or decreasing the retinal slip of borders with a selected surrounding colour was very much smaller than the effect of only having those colours in the surrounding (the classical chromatic induction stimulus). Thus, although the combination of retinal adaptation and eye movements probably does influence chromatic induction to some extent, as was to be expected (Cornelissen & Brenner, 1991, 1995), we can safely conclude that it does not play an important role in chromatic induction.

In Experiment 1, when jittering the image, we did not measure participants’ eye movements, so although we instructed participants to fixate, it remains possible that their eye movements interacted with the motion of the stimulus. For example, they could have followed the jittering stimulus with their eyes to some extent, or moved their eyes slightly in the orthogonal direction to achieve better resolution. Participants may have been able to anticipate the sinusoidal jitter, and to try to move their eyes accordingly despite being instructed not to. However, even if they did, they would probably not have followed the target very smoothly at these high frequencies (Collewijn & Tamminga, 1984; Wyatt & Pola, 1983). Moreover, it is very unlikely that they could meaningfully follow the square wave target motion at 3 Hz, because that would require making six saccades per second at precisely anticipated moments. Thus, if a combination of small fixational eye movements and local adaptation were the driving factor in chromatic induction, we would not expect an 0.2° black rim separating the target from the coloured surrounding to attenuate induction so strongly when the jitter shifted the edges by 1.6°. For the square wave jitter, the frequent abrupt shifts mean that a substantial part of the retina near the target's edge instantaneously switched between being exposed to the colours of the target and of the selected part of the surround at 3 Hz. Thus, the main reservation that we have about the results of the first experiment is that participants’ eyes may have moved substantially in the direction orthogonal to the direction of the jitter to make sure that all edges shift across the retina.

To corroborate our finding with more accurate control of retinal image motion, in Experiment 2 we stabilized selected borders on the retina by moving them contingent to eye movements. Although limits in the precision of eye-tracking and gaze-contingent display control prevent perfect stabilization, this procedure strongly attenuates the image motion normally present on the retina (in the direction in which the image is stabilized). The convergence of the results of the two different approaches provides compelling evidence that the interactions responsible for chromatic induction do not primarily depend on local adaptation and eye movements.

Finding that retinal slip of chromatic borders does not play an important role in chromatic induction is consistent with evidence that simultaneous colour contrast can be strong when presentations are too short for a combination of eye movements and adaptation to play a role (Kaneko & Murakami, 2012; Kaneko, Anstis & Kuriki, 2017). This supports various proposed spatial mechanisms of colour vision (Conway, 2009; Nunez, Shapley & Gordon, 2018). Relying on instantaneous spatial comparisons is advantageous in that it can reduce the sensitivity to the illumination (Land & McCann, 1971; Nascimento et al., 2004; Virsu & Lee, 1983) and to overall changes in the state of adaptation of the retina. But since the same contrast between two surfaces can be achieved with different combinations of colours, one must attribute the contrast between them correctly. Failing to do so could give rise to errors such as those observed in simultaneous colour contrast displays. However, we know that perceived colour does not only depend on the contrasts at surfaces’ borders (Brenner, Granzier & Smeets, 2007b, 2011; Hansen & Gegenfurtner, 2005; Toscani, Gegenfurtner & Valsecchi, 2017; Tyler & Solomon, 2018) and that cells in the visual system do not only respond to spatial colour contrast (Ennis et al., 2014). Thus, our findings do not prove that simultaneous colour contrast is the result of failing to correctly attribute the colour contrast at the border between the target surface and its surrounding. But even if simultaneous colour contrast is the result of misjudging the contrast itself, this is not due to the retinal motion introduced by fixational eye movements.

## Supplemental Material

**Figure:** Movie 1. Impression of what the stimuli in some of the conditions of Experiment 1 looked like.
